# Is Community Relevance Enough? Civic and Science Identity Impact of Microbiology CUREs Focused on Community Environmental Justice

**DOI:** 10.3389/fmicb.2020.578520

**Published:** 2020-12-17

**Authors:** Sarah J. Adkins-Jablonsky, Rob Akscyn, Brad C. Bennett, Qutia Roberts, J. Jeffrey Morris

**Affiliations:** ^1^Department of Biology, University of Alabama at Birmingham, Birmingham, AL, United States; ^2^Department of Biological and Environmental Sciences, Samford University, Birmingham, AL, United States

**Keywords:** civic engagement, community, microbiology laboratory education, science identity, course-based undergraduate research experience

## Abstract

Course-based undergraduate research experiences (CUREs) often involve a component where the outcomes of student research are broadly relevant to outside stakeholders. We wanted to see if building courses around an environmental justice issue relevant to the local community would impact students’ sense of civic engagement and appreciation of the relevance of scientific research to the community. In this quasi-experimental study, we assessed civic engagement and scientific identity gains (*N* = 98) using pre- and post-semester surveys and open-ended interview responses in three different CUREs taught simultaneously at three different universities. All three CURES were focused on an environmental heavy metal pollution issue predominantly affecting African–Americans in Birmingham, Alabama. While we found increases in students’ sense of science efficacy and identity, our team was unable to detect meaningful changes in civic engagement levels, all of which were initially quite high. However, interviews suggested that students were motivated to do well in their research because the project was of interest to outside stakeholders. Our observations suggest that rather than directly influencing students’ civic engagement, the “broadly relevant” component of our CUREs engaged their pre-existing high levels of engagement to increase their engagement with the material, possibly influencing gains in science efficacy and science identity. Our observations are consistent with broader community relevance being an important component of CURE success, but do not support our initial hypothesis that CURE participation would influence students’ attitudes toward the civic importance of science.

## Introduction

Course-based undergraduate research experiences (or CUREs) are becoming increasingly popular in higher education in the United States for their ability to engage university students in authentic research in the context of a course (AAAS, 2009). CUREs are often set apart from more traditional laboratory experiences as allowing students to: (1) learn authentic science practices; (2) discover outcomes not already known to the instructors or students; (3) collaborate; (4) repeat and iterate experiments; and (5) engage with a “broadly relevant” project (Auchincloss et al., 2014). Research results are mixed regarding how much each of these factors contribute to positive student learning outcomes, and not all educators agree that all five factors are necessary parts of every CURE (Ballen et al., 2018). In particular, the “broadly relevant” criterion has come under scrutiny, as it is the most difficult of the five criteria to achieve in practice, particularly at less well-funded institutions. In the meeting report of CUREnet, an online CURE repository, the authors posit “CUREs involve students in work that fits into a broader scientific endeavor that has meaning beyond the particular course context” (Auchincloss et al., 2014). Put another way, broad relevance indicates the presence of, or need for, an outside stakeholder.

Strategies for engaging stakeholders in CUREs vary. The most common solution is to couch the CURE in the context of a larger research project, essentially crowd-sourcing research using student labor (e.g., PARE, Genné-Bacon and Bascom-Slack, 2018; HHMI SEA-PHAGES, Jordan et al., 2014; Tiny Earth, Basalla et al., 2020; and Small World Initiative, Davis et al., 2017) but some effort has been placed into achieving broad relevance through more direct interactions with stakeholders. Ballen et al. (2018) suggested that something as simple as students e-mailing products of their work to an external professor stakeholder could qualify, although Corwin et al. (2018) argue an e-mail may not be sufficient to classify as a broader impact. A wide range of other approaches have also been considered, including acknowledging students in a manuscript, contributing to external databases, and partnering with specific community needs (Corwin et al., 2018). For example, Malotky et al. (2020) paired student research projects like assessing literacy with community-partner engagement such as having students read to an elderly population. These types of examples straddle the line between CUREs and Service-Learning, where course material achieves goals complimentary with direct needs within the community (Smith, 2003; Mendoza et al., 2020).

Student scientific gains in CUREs are also well-documented (Gormally et al., 2009): students i) gain confidence in their ability to do science and to perceive themselves as scientists (Hanauer et al., 2016), ii) are more likely to persist in STEM majors (Estrada et al., 2011), and iii) are more likely to pursue science as a career (Lopatto, 2007). Situated learning theory (Korthagen, 2010) posits that immersion in the cultural milieu of science in the context of authentic research can foster a student’s sense of belonging to the field, but it also suggested to us that broad community relevance in a CUREs might increase feelings of involvement with the impacted stakeholder communities. Sanders and Hirsch (2014) found students who were part of a civically engaged CURE were more likely to want to go into a science career. However, the degree to which broader impacts in CUREs play a role in science identity and persistence gains remains an open question (Corwin et al., 2015, 2018).

Here, we present our results from implementing three different but simultaneous CUREs related to the same environmental justice issue in North Birmingham, Alabama. Since the late 19th century, large-scale industrial activity and political corruption have created a legacy of heavy metal and toxic organic pollution in Birmingham’s 35207 zip code (Allen et al., 2019). In 2012, the evidence of contamination was so significant and widespread that it was declared the 35th Avenue Superfund Site by the Environmental Protection Agency (this site will henceforth be referred to by its zip code, 35207). The residents of 35207 are predominantly African–American, of low socioeconomic status, and suffer from elevated risk of respiratory disease relative to similar populations due to their chronic exposure to toxic heavy metals in their living environment (Allen et al., 2019). As a consequence, understanding how the legacy of pollution influences both the human and environmental health of this area is an issue of social and environmental justice that is directly relevant to members of the greater Birmingham community including all of the students in our study.

The research component of our CUREs focused on various aspects of the co-evolution of heavy metal tolerance and antibiotic resistance in bacteria. Bacteria exposed to either antibiotic or heavy metal stress often evolve resistance to both simultaneously because there is substantial overlap in the genes underlying these phenotypes (Baker-Austin et al., 2006). Thus, as shown in previous contaminated sites (Chen et al., 2019), the possibility exists that long-term exposure to elevated heavy metal concentrations has led to the development of a reservoir of antibiotic-resistant bacteria in the 35207 environment, particularly in the soil. Either through direct infection by environmental microbes, or because of horizontal transfer of resistance genes between environmental and human-associated microbes, 35207 residents could be exposed to elevated risk of recalcitrant antibiotic-resistant infections (Seiler and Berendonk, 2012), although the clinical aspects of this research project are beyond the scope of the student projects described here. Students in our CUREs assessed the degree to which soil bacteria in 35207, compared to a demographically comparable control zip code without elevated heavy metal exposure, were influenced by the history of pollution. Specific research projects are briefly described in the Methods section, and our scientific results will be published in a forthcoming manuscript.

The possible microbiological impact of pollution on human health in 35207 has clear relevance to 35207 residents, and we sought to incorporate that broad community relevance in our courses by i) assigning reading material and videos about 35207 and its residents, ii) incorporating 35207 and data associated with it into lectures, iii) tasking students to generate relevant data, and iv) assigning students to generate products (student-created videos or data) designed to be shared with community stakeholders after the class was completed. These broadly relevant tasks (readings, working with real data, and creating videos) are straightforward educational practices which do not rely on an outside stakeholder during the course and thus could be ideal for CURE educators wishing to incorporate broad relevance in their course. We hypothesized that the inclusion of broader social relevance into the coursework would lead to increases in students’ senses of civic engagement and appreciation for the importance of scientific research for achieving local community goals, while also achieving the increases in scientific efficacy and self-identification commonly observed in CUREs. Specifically, we sought to answer the following questions:

1. To what degree do students at these three universities already agree with statements about civic engagement, science identity, and the role of science in the community prior to their participation in their CURE research project?
2. Are our attempts to include broader community relevance sufficient for students to make gains in their sense of community engagement, and does community engagement interact with gains in science identity and/or science efficacy?

## Materials and Methods

### Preparation

Researchers (SA and QR) collected six homogenized soil samples from public areas in 35207 and six homogenized soil samples from the neighboring control 35214 zip code. 35214 has similar demographics and physical characteristics, but no history of heavy metal pollution. Soil was tested for heavy metal contamination by Sutherland Environmental Testing, which found elevated concentrations of lead, manganese, and zinc in the 35207 zip code areas (data not shown). Approximately 1 g of soil from each public area was given to the teaching teams at the three universities for their analyses; sample IDs were randomized before distribution so that neither students nor instructors would know where a given sample came from.

### Course Descriptions

Participants were recruited during a 14-week Fall 2019 semester from classes at three universities in Birmingham, Alabama. Across all classes, we first identified the CURE learning objectives and research goals based on the recommendations in the CURE literature (Shortlidge and Brownell, 2016; Cooper et al., 2017), which are summarized in Table 1.

**TABLE 1:** Curricular research goals and learning objectives among three non-synchronous CURE courses.

**Course**	**Research goals**	**Student goals**
Microbial Ecology (U1)	1. Discover bacterial taxa that are shared between metal-exposed people and their environments that are absent (or rare) in metal-naive populations 2. Analyze differences in metabolic capacities of metal-exposed soils in comparison with metal-naive soils	1. Master experimental design/scientific method 2. Gain confidence with command-line bioinformatics tools and their interpretation 3. Learn to apply ecological principles to human health questions 4. Collaborate with other student researchers to accomplish a larger task with social relevance
Cell and Molecular Biology (U2)	1. Determine prevalence of metal and antibiotic resistant bacteria from contaminated soil 2. Identify resistant bacteria using morphological, biochemical, and molecular data	1. Apply scientific method to look at the relationship between metal and antibiotic resistance 2. Discuss role of science in social discourse 3. Communicate scientific results
Foundations of Biology (U3)	1. Isolate bacteria from soil environments 2. ID/quantify genes that confer antibiotic/heavy metal resistance in tainted soils	1. Apply modern scientific method to a practical experimental design/interpretation of data 2. Interact with peer group to communicate results

University 1 (U1), the University of Alabama at Birmingham, is a research-intensive public university and doctorate granting institution, Microbial Ecology enrolled 15 students (including both graduate and undergraduate students) for 50-min evening classes Monday, Wednesday, and Friday. Microbial Ecology is cross-listed as an upper division (400-level) undergraduate course as well as a graduate course, with a prerequisite of introductory microbiology. The course was a mixture of lecture, usually on Monday and Wednesday, and bioinformatics workshops, usually on Friday. The lecture portion examined microbial life in natural habitats, such as soil and aquatic ecosystems, with a focus on taxonomic diversity and ecosystem-scale biogeochemical processes. During the workshops, students learned the Standard Operating Procedure for processing next-generation DNA sequence data using mothur (Kozich et al., 2013). Total genomic DNA was extracted from all 12 soil samples with Qiagen PowerSoil kits and used to generate Illumina MiSeq 16S rRNA barcode libraries (Caporaso et al., 2012). In the last month of the semester, U1 students worked in 3-person teams to execute their own bioinformatics projects testing 35207-related hypotheses with this data.

University 2 (U2), Birmingham Southern College, is a private, undergraduate-only liberal arts college. The Cell and Molecular Biology course had an enrollment of 120 undergraduate students. They all attended the same lecture at the same time, however, they were split into six hour-long laboratory sections on Monday and Wednesday mornings. The course served as a 100-level prerequisite for all upper-level biology courses. The teaching team previously pioneered the Metal and Antibiotic Resistance Evolution (MARE) Project, which had students explore the effects of long-term industrial pollution in Birmingham using environmental samples from a Birmingham landmark, Sloss Furnace. Aside from where the environmental samples were collected (North Birmingham residential areas instead of Sloss Furnace), there were no substantial changes to how this course had previously been taught. In their labs, after learning aseptic technique and other fundamental skills (e.g., Gram staining and PCR), students used culture-based methods to screen 35207 and 35214 bacteria for resistance to antibiotics and heavy metals. Agar media had either no addition or contained antibiotics (ampicillin, tetracycline, ciprofloxacin, or a carbapenem drug) or heavy metals (Zn, Mn, and Pb) at concentrations that were previously established to inhibit microbial growth.

University 3 (U3), Samford University, is a private Christian university that awards both undergraduate and graduate degrees. At U3, 48 students across 2 morning sections (24 students per section) took the Foundations of Biology lecture and lab class, which served as a 200-level prerequisite for all upper-level biology courses. The class, composed mostly of freshmen pre-health students and biology majors, met MWF for 110 min per session. The content covered basic biochemistry, cellular metabolism, molecular biology and genetics, taxonomy, and phylogeny, as well as evolution. In the laboratory portion of the course, students began by learning basic microbiology and molecular biology skills. About mid-semester, students worked in 4-person teams to quantify the abundance of total bacteria as well as antibiotic and heavy metal resistance genes in soil-extracted DNA using quantitative PCR (Di Cesare et al., 2016).

In addition to completing the research projects described in Table 1, students in all courses were required to read a news article on the environmental pollution in North Birmingham (Pillion, 2017). Instructors also regularly discussed the importance of the study with students (instructional tools available upon request). Additionally, because student learning objectives in all courses included collaboration and communication, all instructors agreed to include a collaborative video assignment as a part of the course. The video assignments at U1, U2, and U3 were worth 10, 5, and 7.5%, percent of student final grades, respectively. In these assignments, students were tasked with creating 3–5 min instructional videos to share with their student colleagues at the other universities what they had accomplished through their research. These videos needed to also explain the civic engagement component where students contextualized how their specific research aim was related to the environmental justice problem in North Birmingham. Instructors had meetings in-person or through video chat twice a month to provide updates related to education research or curricular progress, including supplies needed for students to carry out their research project.

### Recruitment and Procedure

We surveyed students enrolled in all three courses prior to their first assignment related to experimental design (Table 2) using an online Google survey (see Supplementary Material). We also asked students to self-report their professional goals from a list of academic and non-academic careers at the end of the survey (see Supplementary Material). Students were asked to complete the surveys, outside of class time, 1 week prior to beginning their CURE projects, though students at U1 were given 20 min at the beginning of a class period to incentivize their participation. Students completed the same survey as a post survey in the last week of class. Students at all three universities identified themselves using their student ID numbers to enable pre and post data to be matched as well as to associate survey responses with demographic information (including race, gender, class standing, ACT or SAT equivalent and cumulative GPA) from the university’s registrar. Additionally, a researcher other than the professor assigning grades (either SA or RA) came into each course in the last week of the semester, prior to final exams, to gage students’ attitudes through recorded class discussions using semi-structured interviews. To accommodate the professor and course scheduling, U2 and U3 interviews took place following course-wide review sessions for the final exam and the interviews at U1 took place immediately prior to students taking their final examination. Note that there were two separate course section interviews for U2 but these student comments were combined for purposes of analysis as the instructional curriculum was identical save for the course meeting times. In sum, at U1, eight consenting students took the pre-survey (53% of the class) and two students completed the post surveys (13% of the class). Six U1 students participated in the end of semester interviews. At U2, there were 92 students who consented (77% of the class) and took the pre-surveys and 38 who took the post surveys (32% of the class). 9 U2 students participated in the interviews. In the U3 course, nine students consented (19% of the class) and completed the pre-surveys and five students completed the post surveys (11% of the class). There were 36 total U3 students who were interviewed during two class-wide interviews, one interview with 17 students present and the other with 19 students present. Class discussions were manually transcribed and identifying information was removed.

**TABLE 2:** Procedures across all three CUREs.

**Procedure**	**Length of time required of participants**	**Total # of times procedure performed**
Google surveys (pre and post surveys)	20 min × 2 = 40 min	2 (Pre semester and Post semester)
Discussion interviews	20–50 min	1 (Post semester)

### Instruments

We compiled questions from three Likert-scaled surveys (Box 1) relevant to our learning goals, where lower Likert numbers represent greater levels of agreement with the question. To survey engagement with the community, we used the Civic Engagement Scale (CES), a validated survey for gaging student attitudes and behaviors of engagement related to scholastic service-learning (Doolittle and Faul, 2013). We included CES questions on community mindedness (questions 1, 2, and 6), but excluded questions on volunteering as volunteering was not a focus of any of our courses. To test civic responsibility, we chose the Civic Attitudes about the Relevance of Science (CARS) survey (Siegel and Ranney, 2003) in order to question students’ perceptions of how science shapes attitudes on i) the environment (questions 5 and 42), ii) waste from factories, a specific focus of our courses (question 28), and iii) using evidence to make decisions (question 38). We used questions from the Persistence In The Sciences (PITS) Instrument (Hanauer et al., 2016) to assess Project Ownership Content and Emotion (PITS subsection question 1, abbreviated henceforth as “POCE”); Self-Efficacy (PITS subsection questions 1–5, abbreviated as “SE”), Science Identity (PITS subsection questions 1–4 abbreviated as “SI”) and Scientific Community Values (PITS subsection questions 3 and 4, abbreviated as “SCV”). To account for students from different populations and backgrounds possibly having different ideas about the word community, we chose to ask them to define “community” and “community of scientists” prior to their first use in our survey.

**BOX 1. [t] Survey questions and related acronyms. Full question format is available in Supplementary Material.**

**Civic Engagement Survey and Attitudes about the Relevance of Science**

CES1. I feel responsible for my community.

CES2. I believe I should make a difference in my community.

CES6. I believe that it is important to be informed of community issues.

CARS5. Learning science helps me understand about the environment.

CARS28. Science has nothing to do with local issues, such as waste from nearby factories.

CARS38. Collecting evidence is an important part of making a decision.

CARS42. Knowledge of science will help me protect the environment.

**Persistence in the Sciences (PITS)**

PITS POCE1. My research will help to solve a problem in the world.

PITS SE1. I am confident that I can use technical science skills (use of tools, instruments, and techniques).

PITS SE2. I am confident that I can generate a research question to answer. (POC3)

PITS SE3. I am confident that I can figure out what data/observations to collect and how to collect them.

PITS SE4. I am confident that I can create explanations for the results of the study.

PITS SE5. I am confident that I can use scientific literature and reports to guide my research.

PITS SI1. I have a strong sense of belonging to the community of scientists.

PITS SI2. I derive great personal satisfaction from working on a team that is doing important research.

PITS SI3. I have come to think of myself as a ‘scientist’.

PITS SI4. I feel like I belong in the field of science.

PITS SCV3. I am a person who thinks that scientific research can solve many of today’s world challenges.

PITS SCV4. I am a person who feels discovering something new in the sciences is thrilling.

### Qualitative Procedure and Analysis

In our open interviews with students, we asked them i) the purpose of the course as they understood it, ii) what they learned and their motivations, iii) how the course influenced their career direction, and iv) how their views of science changed. We specifically chose not to prompt students about experimental design, civic engagement, or civic responsibility in order to avoid priming their responses, thus encouraging authentic discussions related to those topics. We used content analysis (Hsieh and Shannon, 2005) to apply the same themes present in the semi-structured interview questions (see Supplementary Material) to categorize the responses, and these overall summaries were decided and agreed upon unanimously by the two coders (RA and SA). Lastly, we used content analysis (Hsieh and Shannon, 2005) to find themes which emerged from student definitions of “community” and came to a complete consensus for coding the students’ definitions.

### Statistical Analysis

Changes in student responses between the pre and post surveys were assessed using linear mixed effects models (deployed in the *lme4* package in R), with university and pre vs post as fixed effects and student ID as a random effect. We note that the incorporation of university as a fixed effect prevents the large difference in sample sizes between universities from skewing model results. Significance of terms was determined by contrasting the full model against nested subset models dropping one of the fixed effects, or the interaction between fixed effects, using the *anova* command in R. Coefficient values reported here are derived from extended marginal means estimates (using the *emmeans* package in R) applied to refined models that eliminate all non-significant terms. All other statistical tests were conducted as linear models in R; as with the linear mixed effects models, the coefficients reported came from extended marginal means estimates from refined models that omit any non-significant predictors.

### IRB Oversight

This study was approved by the IRB Protocols IRB-300004139 (U1), IRB Protocol # 2017-02-011(U2), and IRB Protocol SU-EXMT-A-19-F-2 (U3).

## Results

### Quantitative Data

#### Differences Between Universities

**FIGURE 1:**
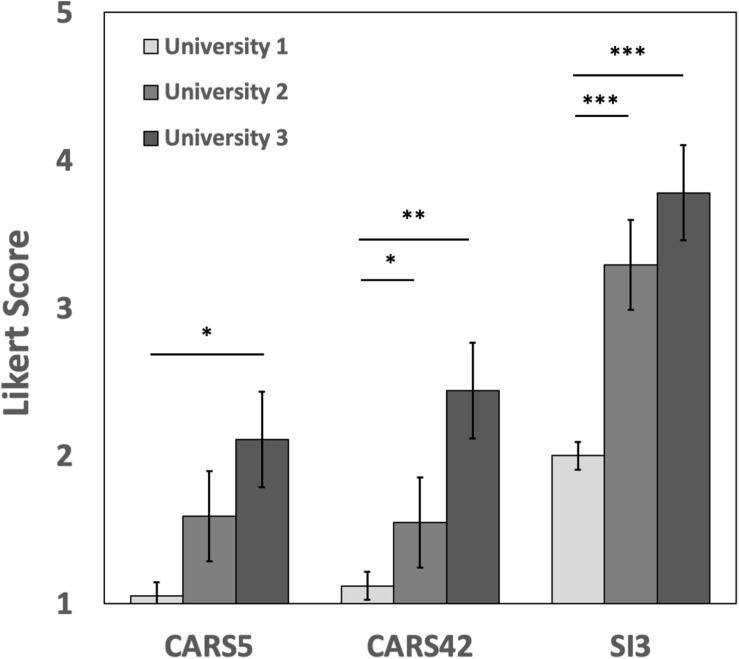
Baseline differences in student attitudes at the three universities. There were only differences in three survey question responses between the universities. Likert scale value 5 indicates strong disagreement and value 1 strong agreement. Error bars in both panels are the 95% confidence intervals from linear model estimates. Asterisks indicate significance levels: ^∗^*p* < 0.05; ^∗∗^*p* < 0.01; ^∗∗∗^*p* < 0.001.

Because of the large differences between the universities and the level of instruction in the different courses, we expected that students would have significantly different baseline attitudes and skills related to our surveys. U1 students reported higher agreement to the CARS statement “Learning science helps me understand about the environment” than U3 students, and were more likely to agree with the PITS statements “I have come to think of myself as a ‘scientist”’ and the CARS statement “Knowledge of science will help me protect the environment” than students at either U2 or U3 (Figure 1). Collectively, these initial differences suggest that the more advanced students of U1 held more professional-like attitudes toward science and the value of science than did the beginning students at U2 and U3. There were no significant between-university differences in any of the other survey questions (data not shown).

We also found that gender significantly influenced student responses in different ways between the three universities we studied. Female students at U1 were more likely than female students at the other universities to agree with belonging to a community of scientists (PITS SI-1), and female students at both U1 and U2 were more likely than female students at U3 to agree with thinking of themselves like scientists (PITS SI-3). On the other hand, U3 males were significantly less likely than males at the other universities to agree with science informing knowledge of the environment (CARS5), the importance of evidence in decision planning (CARS38), and science knowledge informing environmental protection (CARS42). Male students at U3 were also less likely than U3 females to agree with these statements about making a difference in their community (CES2), needing to be informed about community issues (CES6), having satisfaction while working on a science team (PITS SI-2), feeling belonging in science (PITS SI-4), and feeling as though new things in science are thrilling (PITS SCV-4). Further, U3 male students were significantly more likely to agree with science having nothing to do with local issues (CARS Q28) than U3 female or male students from either of the other universities. See Box 1 for statement details. Overall, U3 male students appeared to be strong outliers relative to the rest of the study group; we note however that this was a quite small sample set, consisting of only five male student entries. Importantly, gender did not influence the changes in pre to post for either males or females at any university; the differences described here appear to reflect differences in baseline attitudes and not the effects of the research curriculum on those attitudes.

#### Gains in Civic Engagement and Scientific Identity

**FIGURE 2:**
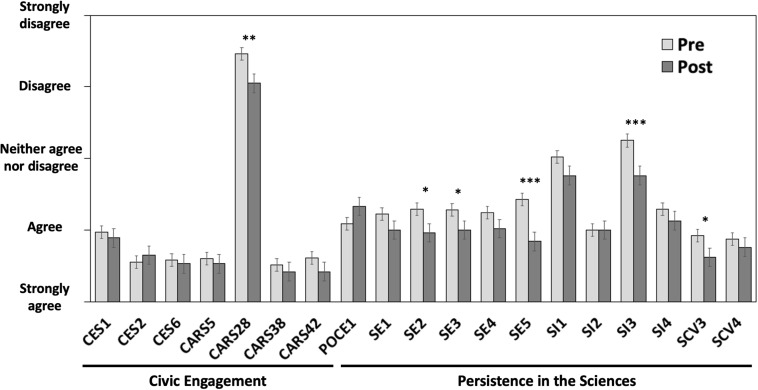
Pre and post responses from 98 students across three simultaneous CUREs. Full questions are provided in Box 1. The Likert scale value 5 indicates strong disagreement and value 1 indicates strong agreement for all questions. Note CARS 28 used an inverted statement. Error bars indicate 95% confidence intervals and asterisks indicate the level of significance from extended marginal means contrasts between pre and post responses derived from linear mixed effects models. ^∗^*p* < 0.05; ^∗∗^*p* < 0.01; ^∗∗∗^*p* < 0.001. There was no significant difference between universities in the change between pre and post responses.

We hypothesized that student agreement to statements on civic engagement would change after a semester of a course focusing on issues of local environmental justice. There was no statistical interaction between university and pre-post change in Likert responses, so the following results represent the entire student population from U1, U2, and U3. Contrary to our expectation, agreement to most of the questions from the CES and CARS instruments did not significantly change over the course of the semester (Figure 2). Only one CARS statement, “Science has *nothing* to do with local issues, such as waste from nearby factories,” changed, but in the opposite direction from our expectation. We were surprised that the average student in the study, after taking a semester of a research course exploring the effects of factory waste, was less likely to agree that factory waste is relevant to science (Figure 2, question CARS28). On the pre-survey, only 2% of students responded in agreement with this statement, whereas on the post survey more than 13% of students agreed. While it is impossible to be certain, we suspect that the reverse wording of this question (i.e., “Science has *nothing* to do with local issues”) may have led to confusion during the post survey; the lack of movement in the “wrong” direction on other questions, and the absence of interview responses supporting such an attitude, provides support for this possibility. The proportion of students in agreement with the remaining CES and CARS questions was very high on both the pre and post surveys. Note that we asked students to define “community” before taking the CES portion of the survey, as ideas about community could differ across student cultures and backgrounds, but themes present in student definitions were not significant predictors of their pre or post responses on relevant questions about community (CES 1, CES 2, CES 6, or PITS-SI 1).

In contrast to our civic engagement questions, we found a significant shift toward agreement on several PITS statements: “I am confident that I can generate a research question to answer”; “I am confident that I can figure out what data/observations to collect and how to collect them”; “I am confident that I can use scientific literature and reports to guide my research”; “I have come to think of myself as a ‘scientist”’; and “I am a person who thinks that scientific research can solve many of today’s world challenges” (Figure 2). These statements include at least one representative from each of the three PITS categories we included: science efficacy, science identity, and scientific community values. As there were no statistically significant differences in the magnitude of change between universities, we conclude that CURE students at all three universities experienced significant gains in the professional virtues measured by PITS, but not in their sense of civic engagement or community responsibility.

### Qualitative Data

We conducted semi-structured interviews with student participants representing each university (U1 *n* = 6, U2 *n* = 9, and U3 *n* = 36) (Table 3). Unsurprisingly, students’ reported understanding of the purposes of their courses and their knowledge gained aligned with the respective course goals (Table 1). Students also consistently brought up the “real world” components of the course as impacting their engagement with the course material. For example, students in the freshmen courses (U2 and U3) but not in the graduate course (U1) explicitly noted their motivation in their research was due to local stakeholders. Students at U1 and U3, but not U2, mentioned being motivated in their research because of the real-world data and student research videos. Example quotes regarding student motivations from U1, U2, and U3 are as follows:

**TABLE 3:** Summary of students’ end-of-semester interview responses to question prompts across three non-synchronous CURE courses.

	**Course**		
**Question prompt**	**Microbial Ecology (U1)**	**Cell and Molecular Biology (U2)**	**Foundations of Biology (U3)**
Purpose	Learn microbial ecology and interactions; Superfund CURE	Heavy metal contamination of soil in Alabama	To engage in real research
Knowledge learned	Diversity of microbial ecology; Bioinformatics pipelines	Effects of heavy metal contamination at the EPA Superfund Site	Molecular biology laboratory techniques
Motivation	Real research and different projects; Increased understanding of Bioinformatics; Research videos seen by other students	Local stakeholders	Real research with unknown solutions with community stakeholders; Research videos seen by other students
Career goals	Affirmed	No changes mentioned	Affirmed or challenged
View of science	Changed; Science depends on many parts and collaboration; Research videos helped explain science projects	No changes mentioned	Changed; No predictive “right” answer; Science does not have to contradict faith

*I think working with real data was a really big part of motivation for this class and I think most of the grad students feel the same way* – U1 student.

*I think it being around the corner from where we are, kinda made us want to know what’s going on. Especially when you heard soil contamination, like what is that exactly? It being so close to you makes you want to dive in and figure out what was going on* – U2 student.

*It made me want to make sure I did it right, like, in chem lab sometimes if you don’t have your data right, you can get numbers from other people if you miss something. But, with this, because it doesn’t matter really, but with this, it’s like you’re actually making an impact on the people who are living in that area and so it made you want to like, do it well and not just do it* – U3 student.

The last two question prompts, about career goals and views of science, were not addressed by students at U2 potentially because the environment in which they were interviewed – right after a study review session – may not have been conducive to a complete and nuanced discourse (RA personal observation). Career goals and views of science shifted for students at U1 and U3. U3 students explicitly mentioned that the student research videos impacted their views of science. In the Supplementary Material, we provide summaries of student responses to each primary question as well as our interview script.

## Discussion

Our primary goal in the work presented here was to investigate whether CURE courses connected to a project with environmental justice implications for the local community would influence student attitudes toward the relationship of science and scientists with the general population and the common good. Secondarily, we wanted to understand how student perceptions of the “broad relevance” of their course research – one of the commonly understood cornerstones of CURE classes – influenced their personal identification as scientists. As we described in greater detail below, our overall conclusion was that our students already had a strong appreciation for the importance of scientific research for society and the community, and this was not changed by our courses. Despite a lack of movement in terms of civic engagement, our students nevertheless became more confident of themselves as scientists, and some of their responses during interviews suggested that the “broad relevance” of their project may have contributed to that improvement.

### Civic Engagement Gains From Surveys and Interviews

Our survey data demonstrated that our students began the course with relatively high levels of community engagement. Pre-scores for CES and CARS showed considerably higher levels of agreement than those for any of our scientific identity metrics (Figure 2). For example, most students across all of the courses either strongly agreed or agreed that they felt responsible for their community, wanted to make a difference in their community, and needed to be informed of community issues. Civic-engagement pedagogy has permeated through education for decades (Putnam, 2000), so it is possible that previous education has played some role in these attitudes. Initially high levels of agreement limited our ability to assess student attitudinal changes in civic engagement using these particular CES and CARS instruments.

Brammer and Morton (2014) were able to tease out civic engagement themes like collaboration, effort, passion, and responsibility from open-ended student reflections in an immersive community-based semester-long public advocacy course. Likewise, our qualitative data provides us a richer story than does the CES and CARS survey results. We expected students in interviews to describe being motivated by the civic engagement parts of the course, including the students’ research being useful for outside stakeholders and creating summary videos about how their research connects to the environmental justice problem. Indeed, students from all three courses reported motivations related to the broader impacts of the CURE (Table 3). Interestingly, we found a marked difference between how the broader impact of the course affected students depending on their course context. Students in the freshmen courses, U2 and U3, emphasized the people and geography of the local stakeholders in their motivations to take their research in the course seriously, whereas the U3 students unanimously expressed that sharing research videos with other scientists was a motivating factor for their continued work on the research projects (Table 3). U1 graduate students were more likely to focus on the scientific impacts of their research as a motivating factor, while still acknowledging the importance of the environmental justice component of the work.

Overall, we found that the broad relevance of projects involving environmental justice and the local community had an effect on student motivation in the course, even though the students had no direct interactions with actual stakeholders in the 35207 community or with other professional scientists working on 35207-related projects. Creating avenues of communication, not just with other student researchers, but with these communities at large, e.g., through Service-Learning pedagogies, could be fruitful improvements for future incarnations of these or similar courses (Smith, 2003).

### Science Identity Gains From Surveys and Interviews

Agreement with several of the statements related to science identity, science efficacy, and scientific values increased from the beginning to the end of the semester (Figure 2). Students across all three universities left their CURE more confident in generating a research question, collecting data, using scientific literature, thinking of themselves as scientists, and thinking research could solve challenges in the world, which are similar to gains reported elsewhere in the literature for CUREs (Gormally et al., 2009; Estrada et al., 2011, Hanauer et al., 2016; Adkins et al., 2018). Many of these results were also corroborated by the interview data (Table 3). Unsurprisingly, all interview responses about the purpose of the course aligned with research goals or learning objectives and corresponded closely with the curricula for each class (Table 1). The fact that similar gains were experienced across very different types of classes, and against very different starting levels of scientific identity and experience, provides further support for the impact of the CURE format on developing scientific confidence in diverse students.

Previous research suggested that civically engaged CUREs might improve scientific persistence (Sanders and Hirsch, 2014), and indeed, this course affirmed career trajectories for several U1 students (Table 3), and some U2 students explained how these new understandings made them want to explore science as an occupation while at least one student explained how the course made them realize scientific research was not for them (Table 3). One benefit of CUREs is that they allow students to have authentic experiences so as to ground these important decisions before entering the job market (Lopatto, 2007). More specifically, U3 students told interviewers they now saw science as a field that is much more open ended than they had understood previously; shifts in science attitudes like this are common outcomes of CUREs (Olimpo et al., 2016).

Finally, we hypothesized that including a conspicuous community relevance component to students’ research would strengthen the “broader relevance” pillar of our CURE, and may therefore have contributed to greater gains in scientific confidence than courses with more abstract or process-oriented research goals. As described above, our hypothesis that our environmental justice focus would increase students’ sense of civic engagement was rejected. However, our interviews clearly showed that students were motivated by the real-world implications of their work, and it is possible that their already high levels of civic engagement underpin that motivation. Thus, instead of our CURE increasing their civic engagement, our students’ civic engagement may have increased the efficacy of the CURE. Our study cannot directly test this possibility, but we suggest that future work should explicitly measure the impact on scientific identity and confidence of increasingly direct interactions with outside stakeholders in CUREs, ranging from the purely procedural (e.g., submitting data to a national database) to the explicitly personal (e.g., Service-Learning).

### Limitations and Future Directions

Our ability to analyze some of our research questions was limited by the fact that not all students completed the entire questionnaire, and in general we had disappointing levels of participation in at least one of our classes. In general, we suspect that the time investment required to complete the entire survey, which included experimental design questions not reported in this manuscript, may have contributed to the relatively low student participation, particularly on the post survey. Some demographic information was also not available for all students, limiting the statistical power of some of our demographic analyses. The size of the student cohorts at the three universities were also quite different, which has the potential to skew aggregate results in favor of the larger U2 cohort, although we attempted to control for this possibility using a linear mixed effects statistical procedure. Moreover, we only surveyed one semester’s worth of classes, and our results may only be representative of students taking these classes in a Fall semester. We acknowledge that this study represents an anecdote in only one city in one country and in one semester, and so more work should be done investigating how broad relevance affects the success of CUREs for a broader demographic of students.

While not including local stakeholders was both situational and intentional, we know this may have limited the ability of the students to appreciate the reciprocal relationship between their scientific work and its human impacts. We are hopeful that we can make this direct interaction a reality in future courses. In particular, we recommend that future attempts to link CUREs to community-relevant projects involve a Service-Learning component. This can enable students to build relationships with local stakeholders, thereby working to have students see science as more than an intellectual pursuit, but also a pursuit that is inextricably linked with human lives.

## Data Availability Statement

The original contributions presented in the study are included in the article/Supplementary Material, further inquiries can be directed to the corresponding author.

## Ethics Statement

The studies involving human participants were reviewed and approved by Institutional Review Boards of UAB, Birmingham Southern College, and Samford University. The patients/participants provided their written informed consent to participate in this study.

## Author Contributions

BB and JM taught courses analyzed in the study. SA, BB, and JM designed the study and IRB protocol. SA and QR assisted in curricula preparation. BB and JM consented students and invited students to participate. SA and RA interviewed students, transcribed interviews, and coded interviews. JM analyzed the Likert data, interpreted the analysis, and created figures. SA, BB, and JM wrote the manuscript with feedback from RA and QR. All authors contributed to the article and approved the submitted version.

## Conflict of Interest

The authors declare that the research was conducted in the absence of any commercial or financial relationships that could be construed as a potential conflict of interest.
