# Supplementary material for: Is Community Relevance Enough? Civic and Science Identity Impact of Microbiology CUREs Focused on Community Environmental Justice
Source: Front Microbiol. 2020 Dec 17;11:578520. doi: 10.3389/fmicb.2020.578520 (PMC7793635; doi:10.3389/fmicb.2020.578520)
Supplement: Supplementary file 2 [file Data_Sheet_2.docx]

**Supplemental Material**

GoogleSurvey Text.... page 1

Interview Summaries.... page 4

**GoogleSurvey Text (administered Pre and Post)**

**Section 1: “Information sheet: An analysis of student outcomes across 3 non-synchronous Course-based Undergraduate Research Experiences”**

*Students were first asked to read over the online information sheet and by submitting the completed survey they consented to participate in this research project.*

**Section 2: University and Identifier**

Which University are you currently enrolled in?

What is you student ID number?

**Section 3: Tool for Interrelated Experimental Design**

*Statements are accompanied by large textboxes*

Use this prompt to answer the following 9 questions:
There is a diversity of feeding/foraging behavior in small freshwater fish called guppies. Some guppies forage closer to their protected refuges, while others travel farther into open water in search of food. You would like to design an experiment to explore the factor(s) that contribute to this difference in behavior.

1. Develop and state a hypothesis for your experiment.

2. Suggest a biological rationale that supports your hypothesis.

3. State the null hypothesis.

4. What are the control group(s)?

5. What are the experimental group(s)?

6. What data will you collect, and how will you collect it?

7. What statistical analysis will you perform to compare the groups?

8. What observations would support your hypothesis?

9. What would support the null hypothesis?

**Section 4: Civic Engagement Survey and Attitudes about the Relevance of Science**

*() Statements are accompanied by Likert 1 (Strongly agree) to 5 (strongly disagree) ovals*

What does the word "community" mean to you?

I feel responsible for my community. (CES1)

I believe I should make a difference in my community. (CES2)

I believe that it is important to be informed of community issues. (CES6)

Learning science helps me understand about the environment. (CARS5)

Science has nothing to do with local issues, such as waste from nearby factories. (CARS28)

Collecting evidence is an important part of making a decision. (CARS38)

Knowledge of science will help me protect the environment. (CARS42)

**Section 5: Persistence in The Sciences**

*() Statements are accompanied by Likert 1 (Strongly agree) to 5 (strongly disagree) ovals*

My research will help to solve a problem in the world. (POC1)

I am confident that I can use technical science skills (use of tools, instruments, and techniques). (POC2)

I am confident that I can generate a research question to answer. (POC3)

I am confident that I can figure out what data/observations to collect and how to collect them. (POC4)

I am confident that I can create explanations for the results of the study. (POC5)

I am confident that I can use scientific literature and reports to guide my research. (POC5)

What does the term "community of scientists" mean to you?

I have a strong sense of belonging to the community of scientists. (SI1)

I derive great personal satisfaction from working on a team that is doing important research. (SI2)

I have come to think of myself as a 'scientist' (SI3)

I feel like I belong in the field of science. (SI4)

I am a person who thinks that scientific research can solve many of today's world challenges. (SCV3)

I am a person who feels discovering something new in the sciences is thrilling. (SCV4)

UNDERGRADUATES ONLY: What are your plans following graduation?

*Mark only one oval*

Ph.D. in biology-related field

Ph.D. in physical science

M.A. in life science

M.A in physical science

Advanced degree in field other than sciences

Medical School (M.D. or D.O.)

M.D./Ph.D.

Other health profession

Law or business degree

Teaching

Military

Peace Corps or similar

Work first

No school after college, science career

No school after college, nonscience career

GRADUATES ONLY: What are your plans following graduation?

*Mark only one oval*

Professional school (medical, dental, etc)

Postdoctoral Researcher

Non-tenure Track Academic

Tenure Track Academic

Science related industry career

Government agency career

Non-research science related career

Non-science related career

**Interview Summaries from each code for U1, U2, and U3**

**Scripted interview questions**

Thank you for taking the time to talk to us about your experiences in your microbiology lab. My name is [*researcher name*]. We want to talk to you because you were a student participant in our study for our qualitative measures, and so now we are looking into more qualitative answers about your experience in this course. This should take about twenty minutes. Please makes sure your phones are silent. Getting into the questions, there are no right or wrong answers, only differing points of view. Feel free to share your point of view if it differs from what others have said. Please keep in mind that we’re just as interested in negative comments as positive comments, and at times negative comments are the most helpful. You’ve probably noticed we are recording. We are doing this because we don’t want to miss any of your comments. People say helpful things in these discussions and we can’t write fast enough to get them all done. Note also that we will omit all names and personal information from the script, which will ultimately go to Dr. [name], Dr. [name], and Dr. [name] after all final grades have been turned in. If at any time you want to stop participating, let us know and we will omit your answers from the data.

*Researchers all asked the following questions in the same sequence, but were able to ask unscripted questions to follow student responses.*

- What is/was the purpose of this course?
- What did you learn in this course?
- Concerning the research aspect of this course, what motivated you to try your best?
- How has this course changed your career direction?
- How did your opinion (or view) of science change during this experience?
- Is there anything else you’d like to tell us about your experience in this class?

**Purpose.**

**U1. Microbial Ecology.** Students agreed that a holistic understanding of microbial ecology and interactions within microbial communities was the purpose of the course. Additionally, the Superfund CURE was cited as a project to give the students direct experience with microbial ecology research.

**U2. Cell and Molecular Biology.** Students indicated that the CURE aspect of the course made them feel more involved in the course and they specifically mentioned heavy metal contamination of soil in Alabama as the course purpose.

**U3. Foundations of Biology.** Students acknowledged the course offered some insight into ‘real world science’, like science methodology and the iterative nature of scientific research, but also expressed a lack of synchronicity between lab activities involving the Superfund CURE and the materials presented in lecture.

**Knowledge learned.**

**U1. Microbial Ecology.** Microbial ecology beyond basic bacteriology, such as virology, metabolism and trophic interactions were described as knowledge gained by these students, in addition to the anthropogenic effects on microbes. The bioinformatics aspect of the course was discussed extensively. Students acknowledged substantial learning gains using the mothur software but also felt technological difficulties were rather cumbersome and may have distracted from learning objectives.

**U2. Cell and Molecular Biology.** U2 students recalled learning about heavy metal contamination through research and about the EPA Superfund site through assigned media articles.

**U3. Foundations of Biology.** Students reported learning molecular biology techniques in laboratory sections as well as large amounts of information in lecture, including taxonomy and cell biology. The volume of work, and the additional work due to the Superfund project, was noted as intensive but it was expressed that the Superfund CURE aspect provided a respite from traditional lecture materials. One student expressed how U3’s learning environment made them more comfortable learning about evolution given their personal religious beliefs.

**Motivation.**

**U1. Microbial Ecology.** The use of real data, the ability to dissect this data with advanced bioinformatic methods, and the making of the summary video for the other two institutions was motivating for several students. Students mentioned that group work in which different groups focused on different questions oriented around a larger central question was enjoyable. There was a consensus on a need to bolster interaction among the partner institutions in future course offerings, and students provided several implementational ideas to consider.

**U2. Cell and Molecular Biology.** Students mentioned the location of the Superfund site and its proximity to their institution as being personally motivating.

**U3. Foundations of Biology.** Students stated that their research potentially having an impact in local communities via a non-traditional lab component and trying to answer ‘real research’ questions without a pre-known answer was motivating. For example, students wanted to refine their laboratory techniques as best as possible because their experiments mattered beyond the classroom. Lastly, having other students, particularly graduate students, view their work through a summation video was especially stimulating for this cohort.

**Career.**

**U1. Microbial Ecology.** A student said the passion of the professor served to strengthen their desire to pursue an academic teaching career. Other students indicated they are still focused on their current research tracks. Another student expressed a slight degree of questioning of career direction but that they had gained an appreciation for collaborative science and the depth of certain areas of study.

**U2. Cell and Molecular Biology.** No comments made were made by these students regarding this topic.

**U3. Foundations of Biology.** Some students said the workload of this course helped them understand and better prepare for continuing in their education and career. Several students expressed affirmation of career direction toward health sciences after taking this course. A student specifically stated the research experience gained in this course will be beneficial in the future as they pursue a STEM career, while another said the course helped them realize a STEM direction may not be what they wish to pursue.

**View of Science.**

**U1. Microbial Ecology.** Students said this course helped them better understand the background involved in scientific research and helped their understanding of larger scientific projects as intensive and collaborative. They also said the short video format provided an opportunity to convey their research to a non-scientific audience. A student said they gained an appreciation for the amount of raw data that can be generated in such a project and the methods used to decipher the information.

**U2. Cell and Molecular Biology.** Student responses did not include anything that was included in the theme changing science views.

**U3. Foundations of Biology**. Students said they found the lab component to be more engaging versus other science courses, like their chemistry labs or high school science courses, and the Superfund aspect further enhanced this lab experience and helped make the scientific process more tangible. They valued doing experiments without a singular, final ‘right’ answer, and while not being able to evaluate their work against predetermined solutions was a bit frustrating, students realized this is often the case when conducting research. A student noted an appreciation for the tendency of scientific endeavors to lead to ‘the more you know, the more you don’t know’ scenarios and the encouraging yet daunting feeling this philosophical realization can induce. Many students could reflect on the nature of science at the end of the semester, but throughout the semester found it difficult to conceptualize the project as a cohesive, stepwise progression, instead finding the process a bit disjointed and hard to connect as the semester progressed. One student said the process of making their video and class presentations helped them understand the bigger concept of the project and recommended that assigning the videos earlier may have helped them understand how lab activities related to the Superfund project.

A few students discussed the benefits of this course in the context of their views of science and their personal faith and how the course fostered personal reflection and growth. They felt this is an important aspect of STEM classes and that perhaps more should be done to encourage such dialogue at least at their private, Christian institution. It was also noted the atmosphere of their university allowed for open dialogue about the interface of religion and science.
